# Household

**Published:** 1889-03

**Authors:** 


### HOUSEHOLD.

Orange Cake.—One cup butter, three cups of sugar, one cup of sweet milk, four and one-half cups of flour, two teaspoonfuls of baking powder and six eggs, saving out the whites of four. Bake in layers. Beat the four whites to a stiff froth and add one pound of powdered sugar. Pare three oranges, and after the frosting is spread over the cake slice the oranges very thin and lay them on the frosting of all the layers except the top.

Oyster Loaves.—With a pointed, sharp knife cut off the tops of some small round French rolls; scrape out the crumbs and fry them crisp in clarified butter; stew as many oysters as needed. First, however, remove the fringe or "beard" and cut them in two. Fill the roll with the oysters, well mixed with the crumbs, add a bit of butter to each, put on the lids and set them in the oven to brown. Serve with fried bread crumbs sprinkled over them.

Creamed Oysters.—For this excellent dish take equal proportions of oysters and cream, say a pint of each, a small bit of onion cut fine, a shred of mace, a tablespoonful of flour, and salt and pepper to taste. Scald the onion and mace in the cream, and the oysters in their own liquor until they curl. Mix the flour with a little cold milk and stir it into the cream when it boils; then skim out the onion and mace from the cream, drain the oysters from their liquor, add them to the prepared cream, and they are ready to be served.

Clam Scallops.—Chop fifty clams fine, and drain off in a colander all the liquor that will come away. Mix this in a bowl with a cupful of crushed cracker, half a cupful of milk, two beaten eggs, a tablespoonful of melted butter, half a tablespoonful of salt, a pinch of mace and the same of cayenne pepper. Beat this into the chopped clams and fill with the mixture clam shells or the silver or stone china shell-shaped dishes sold for this purpose. Bake to a light brown in a quick oven and serve in the shells. Send around sliced lemon with them.

Variety Cake.—Beat together two eggs and one cup of sugar, and three tablespoonfuls of melted butter and one teacupful of sweet milk. Into this stir two cups of flour in which has been well mixed two teaspoonfuls of baking powder. Flavor with lemon, bake in a deep buttered basin that will hold two quarts that it may have room to rise. To make a pudding of it, cut in slices and cover with some nice sauce. Or take the recipe minus the lemon, add one teaspoonful each of ground spices and a spice cake is the result. Or take half of the mixture, add one-half of the spices, place in the pan in alternate layers light and dark, the result is a marble cake; a handful of raisins well rubbed in flour is a nice addition; also a frosting if desired.

Swiss Roll.—Mix one teacupful of flour, the same of finely powdered sugar, and one teaspoonful of baking powder with two eggs (which must not be beaten). Spread this mixture on a buttered baking sheet and bake for seven minutes in a hot oven; then take it from the tin, spread it quickly with jam, and roll it up. Serve either hot or cold with sifted sugar over it.

Fried Chicken.—Fry half an onion chopped fine in a little butter till quite brown. Roll a piece of butter the size of an egg in flour; add to the onion, and in this fry the breast, legs and side bones of the chicken to a delicate brown. Take them out and keep hot in the oven while you add to the sauce in the pan a few mushrooms finely cut up, and salt and pepper to taste; simmer slowly; pour over chicken and serve.

Oyster Soup.—Put into a pan, to heat, two quarts of oysters with their liquor; only let them heat through, and then take them out and add one pint of water, two quarts of milk, one-half pound of butter, and one-half teaspoonful of black pepper and same of all-spice. When the soup is well boiled put in the oysters, having kept them warm in a covered dish. When the oysters are done serve the soup; put in the salt last, as it is likely to curdle the soup.

Bread Omelet.—Take one cup of sweet milk, one cup of fine bread crumbs without crust, a little salt and pepper; beat it all together; add two well-beaten eggs; put in a frying-pan a small lump of butter, let it melt and run all over the pan; now pour in the omelet, cook gently until it sets (about fifteen minutes); loosen the edges and fold one half over the other; now put on a hot plate to fit the pan, hold firmly and turn the pan over; it will come out nice and whole.

Apple Dumplings.—Three teacupfuls of flour, two heaping teaspoonfuls of baking powder, one tablespoonful of butter mixed well through flour, and one teaspoonful of salt. Mix with sweet milk to a dough stiff enough to roll out upon the molding-board. Roll into a sheet half an inch thick and spread with chopped apples. Roll dough up as you would roll rolled jelly cake. Pinch ends well together, so juice cannot escape; place in well-buttered steamer and steam one and one-half hours. Serve with cream or milk and sugar or hard sauce.

Chicken and Egg.—Mince finely any kind of fowl, season to suit the taste of the family. Beat the yolks of three eggs with one-half tablespoonful of flour that has been smoothly mixed with one-half cup of sweet milk, add salt and pepper; beat the whites of the eggs to a stiff foam. Butter an omelet pan well, have it heated, pour in the yolks, put on bits of butter here and there, spread on the minced fowl, and pour the whites over all; let it stand over the fire five minutes then set into the oven to turn a light yellow; slip out carefully and right side upwards on to a well heated platter.

Rye Muffins.—With sweet milk: Three-quarters of a cup of rye meal, three-quarters of a cup of flour, one-half teaspoonful of soda, one teaspoonful of cream of tartar, one tablespoonful of sugar, one saltspoon of salt, one egg beaten and mixed with one-half cup of sweet milk. Put the ingredients together in the order mentioned, and then drop in small spoonfuls into hot fat. Cook until done when tried with a fork. With sour milk: One pint of sour milk, one-half cup of molasses, one saltspoonful of salt, the same of cinnamon, one teaspoonful of soda and two eggs. Add enough rye flour to make a batter that will drop well from the spoon, and then fry in hot lard. These muffins are good eaten plain, with an acid jelly or strong apple sauce; the old-fashioned way was to eat them with cider, but the apple sauce is the best accompaniment.

